# Modules From the End-of-Life Nursing Education Consortium: Japan Core Curriculum Necessary for Second- to Fourth-Year Nurses as Assessed by Advanced Practice Registered Nurses

**DOI:** 10.7759/cureus.51970

**Published:** 2024-01-09

**Authors:** Anri Inumaru, Tomoko Tamaki, Mayumi Tsujikawa, Jun Kako

**Affiliations:** 1 Department of Nursing, Graduate School of Medicine, Mie University, Mie, JPN; 2 Department of Nursing, Shiga University of Medical Science, Shiga, JPN; 3 Faculty of Nursing, Suzuka University of Medical Science, Mie, JPN

**Keywords:** observational study, end of life care, simulation, palliative care, education, nursing

## Abstract

Aim: This study aimed to identify the modules of the End-of-Life Nursing Education Consortium-Japan Core Curriculum (ELNEC-J), which are particularly necessary for second- to fourth-year nurses.

Methodology: This cross-sectional study recruited certified nurse specialists in cancer nursing (CNSCNs) endorsed by the Japanese Nursing Association enrolled in Advanced Practice Registered Nurses (APRNs) in Japan. We asked individuals who were active members of the volunteer association of CNSCNs in the Tokai region to participate via email, and we collected data using Google Forms. The participants were asked about their background, including APRN experience and current position. Furthermore, we asked them to select three necessary modules for second- to fourth-year nurses’ education from the 10 modules of the ELNEC-J.

Results: The study recruited a total of 19 (89%) APRNs (response rate: 100%). Out of them, 14 (73.6%) had more than six years of clinical experience in APRNs, and 12 (63.1%) held managerial positions. Regarding the 10 modules of the ELNEC-J, the responses for the necessary modules were as follows: nursing care at end-of-life 13 (68.4%), pain management 12 (63.2%), symptom management 10 (52.6%), communication 10 (52.6%), and ethical issues in palliative care nursing five (31.6%).

Conclusion: According to the perspective of APRNs responsible for palliative care education for incumbent nurses, nursing care at the end of life, pain management, symptom management, and communication are required for second- to fourth-year nurse education.

## Introduction

In 2002, the World Health Organization (WHO) [1] redefined palliative care as that provided from the early stages of illness and not only at the end of life. In 2007, the Basic Act on Cancer Control was passed in Japan, and the provision of palliative care from the early stages of cancer was identified as one of its priorities. In 2004, the End-of-Life Nursing Education Consortium (ELNEC) developed the ELNEC-Core educational program for nurses in the United States [2]. ELNEC-Japan (ELNEC-J) is the Japanese version of the ELNEC-Core program. In light of the aging population in Japan, the core curriculum for the nurse education program consists of 10 modules, including end-of-life care for the elderly, and this educational program has been provided since 2011. Studies report the educational benefits associated with the program, such as increased knowledge and an improved attitude toward the attainment of quality end-of-life care [3].

According to recent studies, after 10 years, nurses at the national and international levels continue to feel that they lack knowledge of palliative care and are concerned about patient care and communication at the end of life [4-6]. This notion suggests that basic education in palliative care remains insufficiently widespread. Previous studies also mentioned the need to survey nurses to determine what type of education they prefer, and that simulation may be effective in attempting to improve their knowledge and attitudes toward the areas of palliative and end-of-life care [7].

Simulation is an educational strategy that reproduces specific conditions similar to real-life situations to improve knowledge, skills, and attitudes [8]. Simulations related to palliative care are more popular in medicine than in nursing, and communication is evidently frequently addressed as a skill [9]. Nevertheless, a number of studies demonstrate that simulations of palliative care increase the confidence and reduce the anxiety of students [10]. Furthermore, studies on first-year nurses report improved knowledge and skills and high levels of satisfaction [11]. To create a simulation, a needs assessment is first required [12].

Therefore, this study aims to identify the ELNEC-J modules that are considered particularly necessary for second- to fourth-year nurses. ELNEC-J is an end-of-life care education that includes palliative care. Therefore, we first explore the necessary modules within ELNEC-J.

This paper was previously presented as a poster at the 43rd Annual Conference of the Japan Academy of Nursing Science, held on December 3-4, 2022.

## Materials and methods

Study design

The present study adheres to the STROBE Statement checklist of items that should be included in reports of observational studies [13]. This cross-sectional study recruited CNSCNs certified by the Japanese Nursing Association enrolled as Advanced Practice Registered Nurses (APRNs) in Japan. The Institutional Review Board of the Clinical Research Ethics Review Committee of Mie University Hospital approved the study (approval no. U2021-038).

Setting

Data were collected via online surveys using Google Forms from November 8 to 21, 2021.

Participants

The eligibility requirements were as follows: (1) active members of the volunteer association of CNSCNs in the Tokai region of Japan who specialize in palliative care, (2) availability of the Internet to access the online survey. We asked individuals to participate via email. CNSCNs are APRNs responsible for educating nurses within and outside facilities. They also specialize in palliative care, so they are familiar with nurse education in palliative care. Therefore, we deemed that evaluating the necessary modules out of the 10 modules in ELNEC-J is feasible.

Survey items

The participants were asked about their background, including APRN experience and current position. In addition, we asked them to select three necessary modules for second- to fourth-year nurses’ education from the 10 modules of the ELNEC-J curriculum. They completed the questionnaire under the assumption that ELNEC-J participants are nurses responsible for basic palliative care and are equivalent to Clinical Ladder II, which is defined as “able to provide independent nursing care based on a standard nursing plan” [14]. Benner [15] identified this aspect as the “competent” level, which roughly corresponds to the second to fourth years of clinical practice for registered nurses.

Statistical analysis

First, we excluded data with missing values of 20% or more [16] from the analysis. We summarized the characteristics of the participants using descriptive analysis [17] and their responses to the necessary modules as the number of respondents who selected the module and the proportion of selected items divided by the total number of respondents. Statistical analysis was performed using Microsoft Excel 2019 (Redmond, USA). The analysis method was based on Morita et al. [18].

## Results

Characteristics of participants

The study recruited 19 (89%) APRNs (response rate: 100%), out of whom 14 (73.6%) had more than six years of clinical experience in APRNs and 12 (63.1%) held managerial positions. Table 1 presents the descriptive statistics for the complete range of participant characteristics.

**Table 1:** Characteristics of participants APRN: Advanced Registered Practice Nurses

Characteristic		n	%
Years of clinical experience	0-5 years	0	0
	6-10 years	0	0
	Over 11 years	19	100
Years of clinical experience since becoming an APRN	0-5 years	5	26.3
	6-10 years	7	36.8
	Over 11 years	7	36.8
Years of tenure at current workplace	0-5 years	4	21.0
	6-10 years	5	26.3
	Over 11 years	10	52.6
Current job position	Deputy Chief Nursing Officer, Chief of Staff, Section Chief, etc. (management position between Chief of Staff and staff)	10	52.6
	Staff	7	36.8
	Chief Nursing Officer	2	10.5
Place of work experienced as an APRN (Multiple responses allowed)	General ward	13	68.4
	Palliative care team/Outpatient palliative care	12	63.1
	Outpatient chemotherapy room	7	36.8
	General outpatient	6	31.6
	Others	4	21.1
	Medical Cooperation and Nursing Support Office (Consultation Office)	3	15.8
	Palliative Care Unit/Hospice Unit	2	10.5
	Home Care/Visiting Nursing Department	0	0

Necessary modules in ELNEC-J

**Figure 1:**
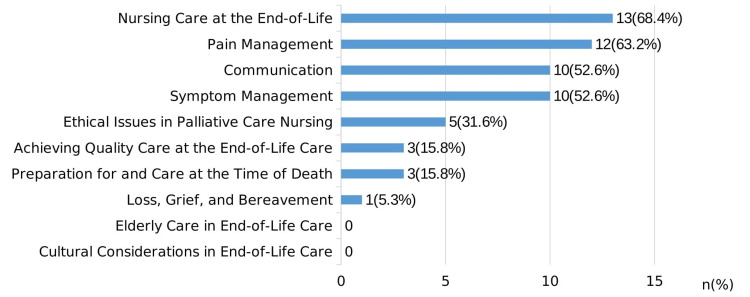
ELNEC-J modules necessary for a competent nurse Participants were asked to choose three modules from 10 modules. This figure was previously presented as a poster at the 43rd Annual Conference of the Japan Academy of Nursing Science, held on December 3-4, 2022.

Regarding the 10 modules of the ELNEC-J, the modules with the greatest number of responses are as follows: nursing care at the end-of-life (13, 68.4%), pain management (12, 63.2%), communication (10, 52.6%), symptom management (10, 52.6%), and ethical issues in palliative care nursing (5, 31.6%). In contrast, the respondents selected neither elderly care in end-of-life care nor cultural considerations in end-of-life care. Moreover, only three (15.8%) selected achieving quality care at the end-of-life care and preparation for and care at the time of death, whereas one (5.3%) opted for loss, grief, and bereavement (Figure 1).

## Discussion

The ELNEC-J modules that APRNs responsible for palliative care education considered necessary were, in order of frequency, nursing care at the end of life, pain management, symptom management, and communication.

Regarding the modules of the ELNEC-J considered necessary by the APRN, the most frequent response was nursing care at the end of life, which included education from the perspective of holistic suffering. A previous study reported that patients with terminal cancer experience total pain [18], and palliative care is holistic and begins with a comprehensive assessment [19]. Therefore, a holistic view of pain is a fundamental principle of palliative care. Nurses must view the topic from the standpoint of total pain. When creating a simulation, it is necessary to take into consideration the total pain.

Patients with incurable cancer require palliative care due to the high prevalence of pain and dyspnea [20], both of which increase in prevalence and severity [21] as the end of life approaches. This notion suggests that modules related to physical pain, such as pain management and symptom management, could also serve as necessary modules. Pain and symptom management have been part of the content of previous simulations [9,11], and the current study further highlights this need.

In a previous study, seasoned professionals identified communication as an important aspect of end-of-life care [22]. Communication with patients and families is essential to palliative care, and education is designed to develop communication skills [23-24]. Communication serves as the basis of palliative care and is the most common content in previous simulation education [9,11]. The current study also takes this perspective into account.

The first limitation of this study is its small sample size; the second limitation is that the survey was restricted to the Tokai region in central Honshu, Japan. Therefore, it is possible that different needs may emerge if different populations are surveyed. The third limitation is that this study did not recruit nurses who were nonspecialists in palliative care to assess palliative care education needs. It is possible that different needs could be identified by also surveying the needs of generalists.

## Conclusions

This study aimed to identify the ELNEC-J modules that are considered particularly necessary for second- to fourth-year nurses. According to the perspective of APRNs responsible for palliative care education for second- to fourth-year nurses, nursing care at the end of life, pain management, symptom management, and communication are required for their education. The results of this study may contribute to the development of palliative care education programs.
